# Exogenous 24-Epibrassinolide Enhanced Drought Tolerance and Promoted BRASSINOSTEROID-INSENSITIVE2 Expression of Quinoa

**DOI:** 10.3390/plants13060873

**Published:** 2024-03-18

**Authors:** Ya-Li Zhou, Xin-Yong You, Xing-Yun Wang, Li-Hua Cui, Zhi-Hui Jiang, Kun-Peng Zhang

**Affiliations:** 1 College of Biological and Food Engineering, Anyang Institute of Technology, Anyang 455000, China; zhouyali0516@163.com (Y.-L.Z.); xinyong8206@163.com (X.-Y.Y.); jiangzhihui19870326@126.com (Z.-H.J.); zhangkunpengag@163.com (K.-P.Z.); 2 College of Mechanical and Electronic Engineering, Northwest A&F University, Yangling 712100, China; cuilihua0320@163.com

**Keywords:** drought stress, brassinosteroids, VIGS, *CqBIN2*, quinoa, transcriptomics

## Abstract

Brassinosteroids (BRs) are involved in the regulation of biotic and abiotic stresses in plants. The molecular mechanisms of BRs that alleviate the drought stress in quinoa have rarely been reported. Here, quinoa seedlings were treated with 24-epibrassinolide (EBR) and we transiently transferred *CqBIN2* to the quinoa seedlings’ leaves using VIGS technology to analyze the molecular mechanism of the BR mitigation drought stress. The results showed that EBR treatment significantly increased the root growth parameters, the antioxidant enzyme activities, and the osmolyte content, resulting in a decrease in the H_2_O_2_, $O_{2}^{∙-}$, and malondialdehyde content in quinoa. A transcriptome analysis identified 8124, 2761, and 5448 differentially expressed genes (DEGs) among CK and Drought, CK and EBR + Drought, and Drought and EBR + Drought groups. WGCNA divided these DEGs into 19 modules in which these characterized genes collectively contributed significantly to drought stress. In addition, the EBR application also up-regulated the transcript levels of *CqBIN2* and proline biosynthesis genes. Silenced *CqBIN2* by VIGS could reduce the drought tolerance, survival rate, and proline content in quinoa seedlings. These findings not only revealed that exogenous BRs enhance drought tolerance, but also provided insight into the novel functions of *CqBIN2* involved in regulating drought tolerance in plants.

## 1. Introduction

Quinoa (*Chenopodium quinoa* Willd.) is the only monoculture plant that meets the basic nutritional needs of humans and is an important crop to improve global food security [1]. In recent years, the production and consumption of quinoa have increased considerably due to its great nutritional value, including protein, essential amino acids, polysaccharides, unsaturated fatty acids, trace elements, etc., polyphenols, flavonoids, and saponins [2]. Phenolic compounds have strong antioxidant activity and scavenging free radicals [3], with preventive effects against chronic diseases, including cardiovascular diseases, cancer, and diabetes mellitus [4]. Furthermore, quinoa has been widely cultivated worldwide due to its high genetic variability and provides the necessary resistance to harsh environments. However, the escalating frequency of drought events, attributable to global climate change, presents an ongoing peril to quinoa production. To improve the nutritional composition of human diets, it is imperative to ensure the sustainable availability of quinoa. Consequently, the enhancement of the tolerance of quinoa to drought assumes paramount importance, as it enables adaptation to evolving environmental conditions and the attainment of consistently elevated yields.

BRs are a class of natural steroid hormones that regulate plant growth and development and promote growth and enhance plant resistance to biotic and abiotic stresses [5,6,7]. The contribution of exogenous BR treatments to drought tolerance has been studied in many plants. In wheat, the exogenous application of 24-epibrassinolide could mitigate drought stress by enhancing antioxidant enzyme activities and improving the level of abscisic acid, indol acetic acid, and cytokinin [8]. Khamsuk et al. [9] demonstrated that exogenous BR could maintain cellular osmotic balance, improve the water status of chili pepper leaves, and alleviate the negative effects of drought on the physiological responses of plants under severe water stress. Exogenous BR treatment could activate the activity of antioxidant enzymes and the content of proline, an osmoregulatory substance, mitigating the inhibitory effects of drought stress on plants [10,11].

Meanwhile, the overexpression of BR biosynthesis genes in plants can also enhance plant drought tolerance, such as the overexpression of *AtDWF4* in oilseed rape and *SoCYP85A1* in spinach, both of which enhanced drought resistance in plants [12,13]. Similarly, the gene expressions during BR signaling contribute to improving plant resistance to drought. In maize, ZmBSK1 interacts with ZmCCaMK, calcium-dependent protein kinase, and phosphorylates ZmCCaMK and plays an important role in the regulation of maize tolerance to drought stress [14]. *TaBZR2* overexpression enhances drought tolerance in transgenic wheat, while drought tolerance was reduced when the expression level was down-regulated by RNA interference; furthermore, the TaBZR2 transcription factor activates the expression of the glutathione transferase *TaGST1* and mediates crosstalk in the BR and drought signaling pathways to improve drought tolerance in wheat [15].

In BR signaling, BRASSINOSTEROID-INSENSITIVE2 (BIN2) is an important GSK3-like kinase. BIN2 is a negative regulator and acts by phosphorylating two transcription factors BRASSINAZOLE-RESISTANT 1 (BZR1) and BRI1-EMS-SUPPRESSOR1 (BES1) [16]. BIN2 can phosphorylate the NAC family transcription factor RD26 to enhance drought tolerance signaling pathways in Arabidopsis [17]. BIN2 phosphorylates and stabilizes the AP2/ERF transcription factor TINY to positively regulate drought tolerance while inhibiting plant growth; under optimal growth, BIN2 promotes the degradation of the TINY protein to prevent its activation of stress responses that affect crop growth [18]. BIN2 phosphorylates ICE1 in response to salt stress mediated by abscisic acid (ABA), thus negatively regulating its stability in response to cold stress [19]. Meanwhile, BIN2 can be dephosphorylated by ABA INSENSITIVE1 (ABI1) and ABI2 in the ABA signaling pathway, and ABA activates BIN2 by inhibiting the activities of ABI1 and ABI2, after which BIN2 phosphorylates SnRK2s and activates downstream signaling pathways to regulate the opening and closing of plant stomata and the efficiency of water use [20]. These studies verified that BIN2 plays a crucial role in the crosstalk between multiple signaling pathways in the regulation of stress tolerance. However, in BR signaling, it is not clear whether and how BIN2 is involved in drought stress in quinoa.

This study aims to reveal the effect of exogenous EBR applied to alleviate oxidative damage and to improve drought tolerance in quinoa seedlings by determining antioxidant enzyme activities, osmolyte content, endogenous hormones, and genes’ mRNA expression levels. It clarified the effects of EBR on *CqBIN2* and proline biosynthesis and provided a theoretical basis for further research on the molecular mechanisms of *CqBIN2* in alleviating drought stress in quinoa. The present study provides theoretical support for the application of EBR to alleviate the damage of drought stress in quinoa.

## 2. Materials and Methods

### 2.1. Plant Materials and Growth Conditions

Quinoa seedlings were grown in a climatic chamber at 25 °C with a 16 h/8 h light/dark cycle. Quinoa seeds were planted on a seedling tray (50 mm × 50 mm × 50 mm). The seeds were sown at each point, and when the seedlings grew two cotyledons, they were transferred to seedling pots (90 mm × 90 mm × 100 mm), and finally, one seedling was planted in each seedling pot. Before drought treatment, all quinoa seedlings were routinely managed in a climate chamber and fertilized with Hoagland nutrient solution. Humus soil and perlite were mixed in a 3:1 ratio as substrates for the cultivation of quinoa seedlings. Before drought treatment, all quinoa seedlings were irrigated with 1/2 Hoagland’s solution to 70% of substrates’ water holding capacity (WHC).

### 2.2. ERB Treatment and Drought Stress

When the quinoa grew six cotyledons, quinoa seedlings with a uniform growth status were selected for EBR and drought treatment. The 24-epibrassinolide (EBR) was procured from Sigma (Sigma, St. Louis, MO, USA) and dissolved in absolute ethanol to achieve final concentrations of 0.25, 0.5, 1, 2, and 2.5 μM, respectively. All quinoa seedlings were sorted into twelve groups: normal watering (70% WHC) (CK1), drought treatment (30% WHC) (CK2), different concentrations (0.25, 0.5, 1, 2, and 2.5 μM) of EBR + normal watering (70% WHC), and different concentrations (0.25, 0.5, 1, 2, and 2.5 μM) of EBR + drought treatment (30% WHC). Each group was divided into three replicates, each with no less than 10 seedling pots. For the EBR + normal watering and EBR + drought treatment groups, varying concentrations of EBR were applied evenly to cover the entire surface area of the quinoa seedlings. We then investigated the effects of these treatments on the morphology, leaf water content, antioxidant enzyme activity, osmoregulatory substance content, and expression levels of related genes in quinoa plants under drought stress. Through quantitative and qualitative analysis, we determined the optimal EBR concentration for alleviating drought stress in quinoa. Our findings revealed that the 0.5 μM EBR treatment demonstrated the most effective results in mitigating drought stress in quinoa plants. Thus, this research primarily focused on the outcomes of the 0.5 μM EBR treatment, with 0.5 μM EBR + normal watering and 0.5 μM EBR + drought treatment recorded as EBR1 and EBR2, respectively. In addition, the gene was transiently transformed into quinoa leaves using VIGS technology without adding water to simulate drought stress. Each treatment contained 30 quinoa seedlings. During drought treatment, the morphology of the quinoa seedlings was observed. The roots and leaves of the quinoa seedlings were collected at 0, 1 d, 2 d, 3 d, 6 d, and 9 d, and stored at −80 °C.

### 2.3. Scan of Roots

The root morphology parameters (length, surface area, volume, average root diameter, and number of lateral roots) of the quinoa seedling were scanned by an Epson Expression 1680 flatbed scanner (Epson, Long Beach, CA, USA). Specifically, after taking leaves at 0, 1, 2, 3, 6, and 9 days of drought stress, quinoa seedlings were removed from the soil, and the roots were cleaned with distilled water to wash off the soil. A transparent tray was filled with 12–17 mm of water to submerge the roots, and the roots were adjusted so that they did not become entangled with each other and scanned using a scanner. Furthermore, the root morphology parameters were obtained using WinRHIZO Pro2007 system software (Regent Instruments, Québec City, QC, Canada) to analyze the root conformation.

### 2.4. Determination of Lipid Peroxidation

Malondialdehyde (MDA) was determined according to the colorimetric method of thiobarbituric acid. An amount of 1 g of fresh leaves of quinoa was homogenized in 6 mL 10% trichloroacetic acid (TCA) and centrifuged for 5 min; the supernatant was the MDA extract. The 2 mL supernatant (control added 2 mL distilled water) was taken, 2 mL 0.6% thiobarbituric acid solution (TBA) was added, the mixture was placed in a boiling water bath for 15 min, followed by rapid cooling on ice, and then centrifuged at 10,000× *g* for 10 min at 4 °C. Finally, the absorbance of the supernatant was measured at 532 nm, 600 nm, and 450 nm.

The production rate of superoxide ($O_{2}^{∙-}$) and H_2_O_2_ was determined using the detection kits H_2_O_2_ (BC3590, BC1290, Beijing Solarbio Science & Technology, Beijing, China).

### 2.5. Determination of Soluble Protein, Free Proline, and Antioxidant Enzyme Activities

Soluble protein contents were determined as described by Bradford [21] with minor modifications. Soluble protein contents were calculated by bovine serum albumin as a calibration standard. The content of proline (BC0290) and the activities of superoxide dismutase (SOD, BC0175), catalase (CAT, BC0205), peroxidase oxidase (POD, BC0090), ascorbate peroxidase (APX, BC0220), glutathione reductase (GR, BC1160), monodehydroascorbate reductase (MDHAR, BC0650), and dehydroascorbate reductase (DHAR, BC0660) were determined by the manufacturer’s instructions (Beijing Solarbio Science & Technology, Beijing, China).

### 2.6. BR and ABA Content

An amount of 0.5 g of crushed leaves samples was extracted in 3 mL of extraction solution (which contains 80% methanol, 0.02% 2, 6-2 tertiary butyl-4-methyl phenol, and 0.05% citric acid monohydrate) for 4 h at 4 °C, 6 mL of dichloromethane was added, and the mixture was extracted for l h at 4 °C. The mixture was centrifuged at 10,000 rpm at 4 °C for 10 min. The substrate freeze-dried the bottom liquid with a freeze dryer, and then 1 mL extraction solution was added to dissolve the dried product for HPLC analysis.

### 2.7. Transcriptome Sequencing and Data Analysis

The raw reads were transformed from the sequencing raw image data by CASAVA base recognition. To obtain high-quality data, raw reads were filtered, that is, splice reads, low-quality reads, and low-quality reads with a proportion of unknown base information ≥ 5 or Qphred ≤ 20 bases were removed. The GC content of clean reads was calculated. The Q20 and Q30 values were also produced by FastQC to evaluate the base quality. The clean reads were mapped to the hickory reference genome using HISAT2 with default parameters. Gene expression levels were determined using the RPKM (reads per kb per million reads) method. Gene expression differences in the |fold change| ≥ 2 and false discovery rate (FDR) < 0.05 criteria were identified as DEGs for drought-stressed samples compared to the control, with positive values of |fold change| being up-regulated genes and negative values being down-regulated genes. Furthermore, GO functional enrichment analysis and KEGG metabolic pathway analysis were performed on DEGs to identify the GO entries that were significantly enriched in DEGs and the main metabolic pathways in which they were jointly involved. The gene co-expression networks were constructed with the WGCNA based on the FRKM values of all DEGs.

### 2.8. RNA Extraction and Quantitative Real-Time PCR (qRT-PCR)

Total RNA was extracted and reverse-transcribed according to the manufacturer’s instructions (R401-01, R233-01, Nanjing Vazyme Biotech Co., Ltd., Nanjing, China). And the cDNA was diluted to 200 ng μL^−1^ with sterile enzyme-free water. qRT-PCR was performed with the Bio-Rad IQ5 real-time PCR system. The primers were designed by Primer 5 (Supplementary Materials Table S1). The gene expression level was determined using a two-step RT-qPCR kit (Vazyme Biotech Co., Ltd., Nanjing, China) operated according to the manufacturer’s instructions. The $2^{-∆∆Ct}$ method was used to calculate the relative transcription level normalized by *CqMON1*.

### 2.9. Virus-Induced Gene Silencing (VIGS) of Quinoa Seedlings

*CqBIN2* was silenced by VIGS in quinoa seedlings through BSMV vectors, which were introduced into the quinoa cotyledon by *Agrobacterium tumefaciens* injection. A 461-bp CqBIN2-specific fragment was obtained by the primer amplification of *CqBIN2*-BSMV-F/R. The CqBIN2-specific product of PCR was digested with BamHI and KpnI restriction enzymes. A 461-bp specific fragment of CqBIN2 was recombined with a linearized BSMV vector fragment using homologous recombination, and the recombinant plasmid was named BSMV-*CqBIN2*, the γ empty plasmid was a negative control. In vitro, the transcription of the different plasmids was performed according to the kit instructions (Ribo MAX™ Large Scale RNA Production System and Ribo m7G Cap Analog Kits (Promega, Madison, WI, USA)). The names and sequences of the primer pairs for VIGS are given in the Supplementary Materials Table S2. The seedlings were incubated in conservatories (16 h light/8 h dark, 25 °C and 60% humidity conditions).

### 2.10. Statistical Analysis

All determinations were performed in triplicate. The data were analyzed using a one-way analysis of variance (ANOVA) with IBM SPSS Statistics 24.0 software (IBM Corporation, Armonk, NY, USA). Statistical significance was set at 0.05. All results were means of three replicates with ±standard deviation (SD), and different lowercase letters (a, b, c, d) in the figures indicate a significant difference between treatments. All figures were drawn using OriginPro 2018 (OriginLab, Northampton, MA, USA).

## 3. Results

### 3.1. Growth Parameters

**Figure 1:**
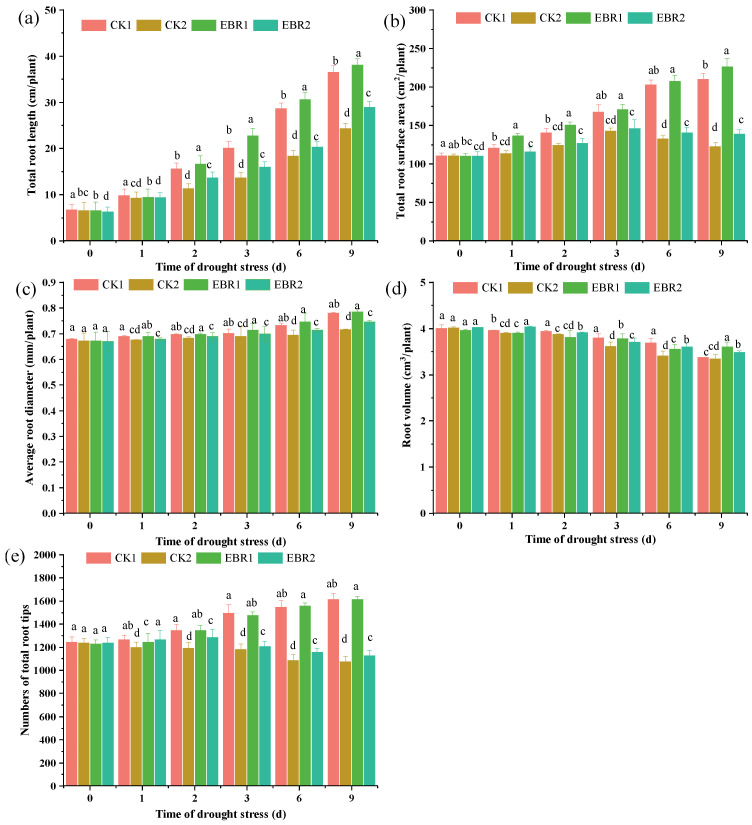
Effects of drought stress on the root growth parameters of quinoa seedlings. (**a**): Total root length, (**b**): Total root surface area, (**c**): Average root diameter, (**d**): Root volume, and (**e**): numbers of total root tips. Values are means ± SD (*n* = 3); different lowercase letters indicate significant differences between treatments (*p* < 0.05, Duncan’s multiple range test). CK1: normal watering; CK2: drought stress; EBR1: EBR + normal watering; EBR2: EBR + drought stress.

Plant parameters decreased considerably with drought stress compared to normal watering (Figure 1). Total root length decreased by 3.48~56.03% in CK2 plants compared to CK1 plants. The total root surface area decreased by 6.32~71.31% in CK2 plants over CK1 plants. Compared to CK2, EBR2 treatment significantly increased root morphology parameters. Compared to CK2 plants, the total length of the roots increased by 3.54~17.21% under EBR2 treatment and also reached the maximum value of 17.21% at 2 d (Figure 1a) and about a 1.68~11.49% and 0.42~3.97% increase in total root surface area and total root diameter, respectively (Figure 1b,c). There was a gradual downward trend in root volume under different treatments (Figure 1d). In response to drought stress, root tip number was significantly increased by 2.07~7.16% in EBR2 plants, compared to CK2 plants (Figure 1e).

### 3.2. H_2_O_2_, $O_{2}^{∙-}$, and MDA Content

**Figure 2:**
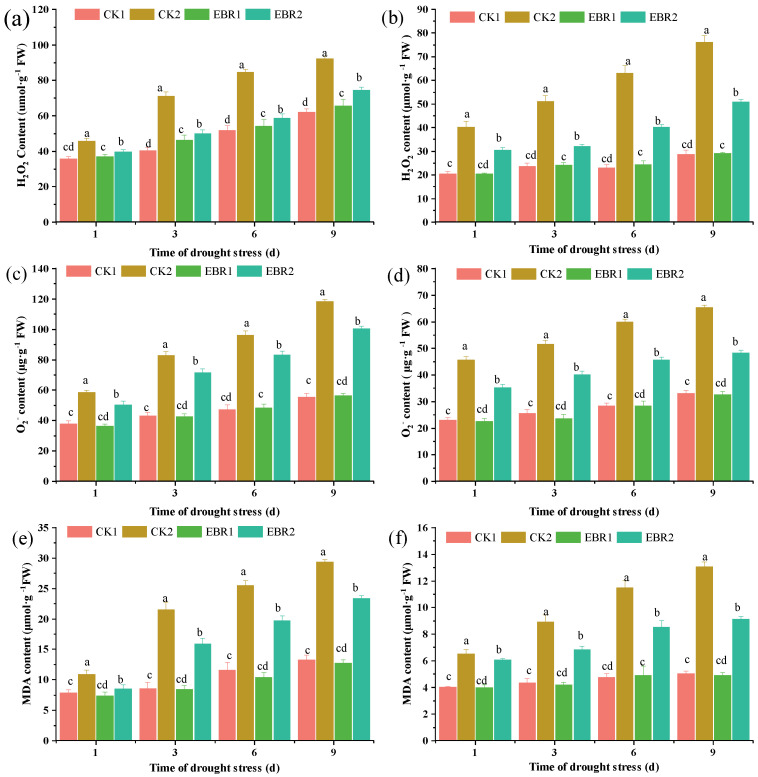
Effects of drought stress on the content of H_2_O_2_, $O_{2}^{∙-}$, and MDA in quinoa seedlings’ roots (**a**,**c**,**e**) and leaves (**b**,**d**,**f**). Values are means ± SD (*n* = 3); different lowercase letters indicate significant differences between treatments (*p* < 0.05, Duncan’s multiple range test). CK1: normal watering; CK2: drought stress; EBR1: EBR + normal watering; EBR2: EBR + drought stress.

EBR treatment significantly reduced the generation rate of H_2_O_2_, $O_{2}^{∙-}$, and MDA contents both in the roots and leaves of quinoa seedlings. The H_2_O_2_ content in the EBR2 plants was greatly reduced by 15.20~44.44%, and 31.63~59.03% in the CK2 plants in roots and leaves, respectively (Figure 2a,b). Under drought stress (CK2), the contents of $O_{2}^{∙-}$ in roots and leaves were significantly increased by 35.17~53.23% and 49.35~52.55%, respectively, compared to CK1. However, the $O_{2}^{∙-}$ production was reduced by 15.73~17.90% and 28.36~35.55% in the roots and leaves of EBR2 plants compared to the CK2 plants (Figure 2c,d). Compared to the CK2 plants, the MDA content in EBR2 plants was reduced by 25.65~34.97% and 8.09~43.05% in roots and leaves, respectively. Furthermore, the MDA content decreased to a minimum value of 34.97% at 3 d in the roots and by 43.05% at 9 d in the leaves (Figure 2e,f). Additionally, the EBR2 plants exhibited a slight wilting phenotype, which was not significantly different from the CK1 plants. These results suggest that exogenous EBR was involved in membrane lipid peroxidation induced by the accumulation of reactive oxygen species, mitigating the degree of damage to the membrane system and alleviating drought damage in quinoa.

### 3.3. Soluble Protein and Proline Content

**Figure 3:**
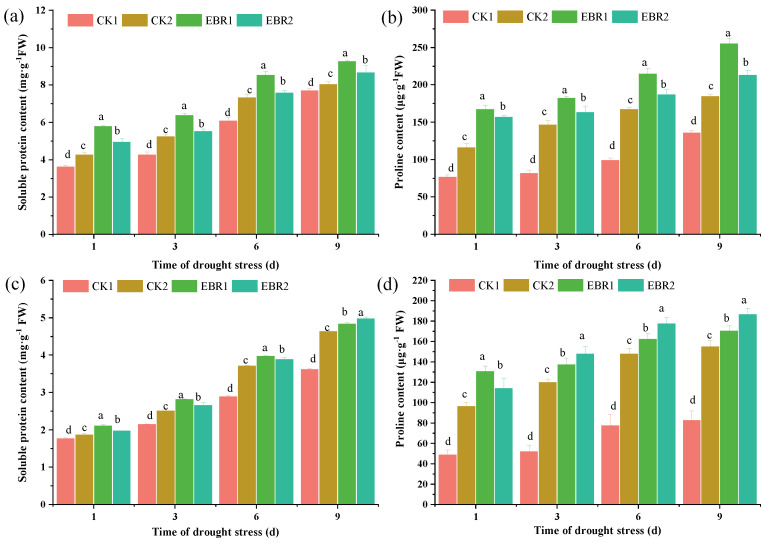
Effects of drought stress on the content of soluble protein and proline in roots (**a**,**c**) and leaves (**b**,**d**). Values are means ± SD (*n* = 3); different lowercase letters indicate significant differences between treatments (*p* < 0.05, Duncan’s multiple range test). CK1: normal watering; CK2: drought stress; EBR1: EBR + normal watering; EBR2: EBR + drought stress.

The value of soluble protein and proline in roots and leaves increased significantly with either drought or EBR treatment over normal watering. Compared to CK2 plants, EBR2 treatment led to a significant increase in soluble protein contents and reached the highest level, an increase of 71.85% and 67.41% at 9 d in roots and leaves, respectively (Figure 3a,c). The proline content of the EBR treatment plants (EBR1 and EBR2) was dramatically increased and significantly higher than that of the CK1 and CK2 plants. The proline content gradually increased and reached the maximum recorded in EBR2 plants, a maximum increase of 13.23% in roots at 9 d and 18.92% in leaves at 3 d (Figure 3b,d), over the CK2 plants.

### 3.4. Antioxidant Enzyme Activities

**Figure 4:**
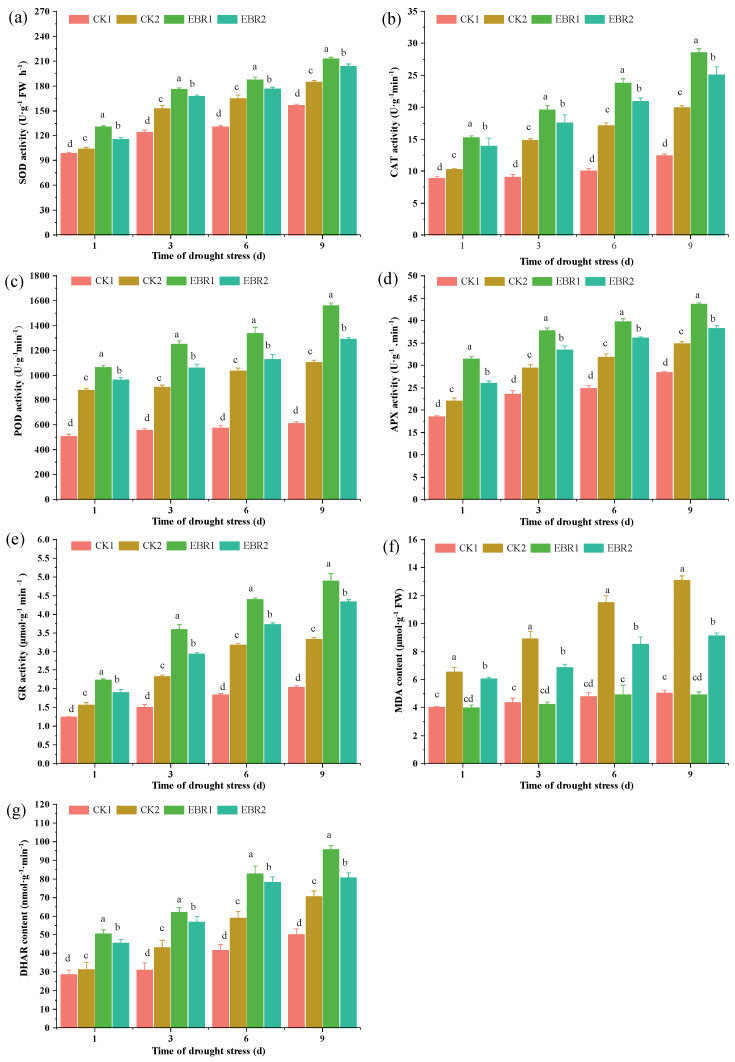
Effect of drought stress on the content of antioxidant enzymes in leaves. (**a**): SOD, (**b**): CAT, (**c**): POD, (**d**): APX, (**e**): GR, (**f**): MDA, and (**g**): DHAR. Values are means ± SD (*n* = 3); different lowercase letters indicate significant differences between treatments (*p* < 0.05, Duncan’s multiple range test). CK1: normal watering; CK2: drought stress; EBR1: EBR + normal watering; EBR2: EBR + drought stress.

The antioxidant enzyme (SOD, CAT, POD, APX, GR, MDHAR, and DHAR) activities were increased in the presence of drought and EBR (Figure 4) and reached the maximum value in EBR1 plants. Compared to CK2, the SOD activity was much higher than in the EBR2 plants, increasing by 9.04~10.44% (Figure 4a). CAT activity was enhanced under drought stress, whereas it was further enhanced with EBR treatment. Compared to CK2, CAT activity in EBR2 plants increased by 15.92~26.58% (Figure 4b). A significant increase of 7.92~14.86% observed in POD activity was observed in EBR2 plants, and the percentage reached maximum at 3 d (Figure 4c), compared to CK2 plants. EBR and drought stress induced a great and continuous increase in the activities of APX, GR, MDHAR, and DHAR, indicating that the antioxidant response was enhanced. There was an increase in APX, MDHAR, and DHAR activity up to 9.08~14.98%, 3.99~5.82%, and 12.50~31.51%, respectively, in EBR2 plants over CK2 plants (Figure 4d,f,g). GR activity in EBR2 plants reached a maximum value of 23.21% at 9 d, compared to CK2 plants (Figure 4e).

### 3.5. ABA and BR Content

**Figure 5:**
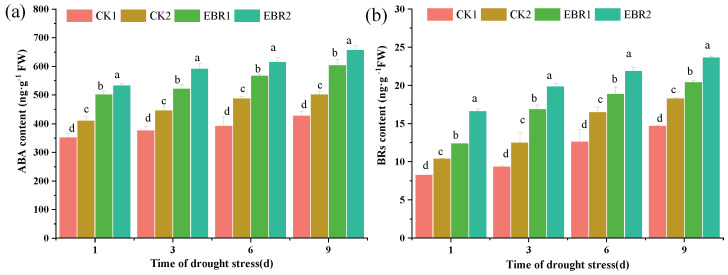
Effects of drought stress on the content of ABA (**a**) and BR (**b**) in quinoa seedlings. Values are means ± SD from Duncan’s multiple range test. Values are means ± SD (*n* = 3); different lowercase letters indicate significant differences between treatments (*p* < 0.05, Duncan’s multiple range test). CK1: normal watering; CK2: drought stress; EBR1: EBR + normal watering; EBR2: EBR + drought treatment.

EBR application can significantly increase BR and ABA contents, further increasing the drought resistance of the quinoa seedlings (Figure 5). ABA accumulation is one of the most rapid responses to abiotic stress, especially drought stress. Compared to CK1 plants, drought stress (CK2) significantly increased ABA content by 14.16~19.77% and reached a maximum value of 19.77% at 6 d. Furthermore, compared to CK2 plants, EBR2 treatment increased the ABA content by 20.70~24.54% and reached a maximum value of 24.54% at 3 d. The BR content in the leaves of quinoa significantly increased the drought resistance of quinoa. Compared to CK1 plants, drought stress (CK2) significantly increased the BR content by 19.87~25.06%. Compared to CK2, the BR content was increased by 22.66~24.78% in the EBR2 plants. These results indicate that exogenous EBR treatment promoted the accumulation of endogenous hormones ABA and BR.

### 3.6. Transcriptomic Analysis

**Figure 6:**
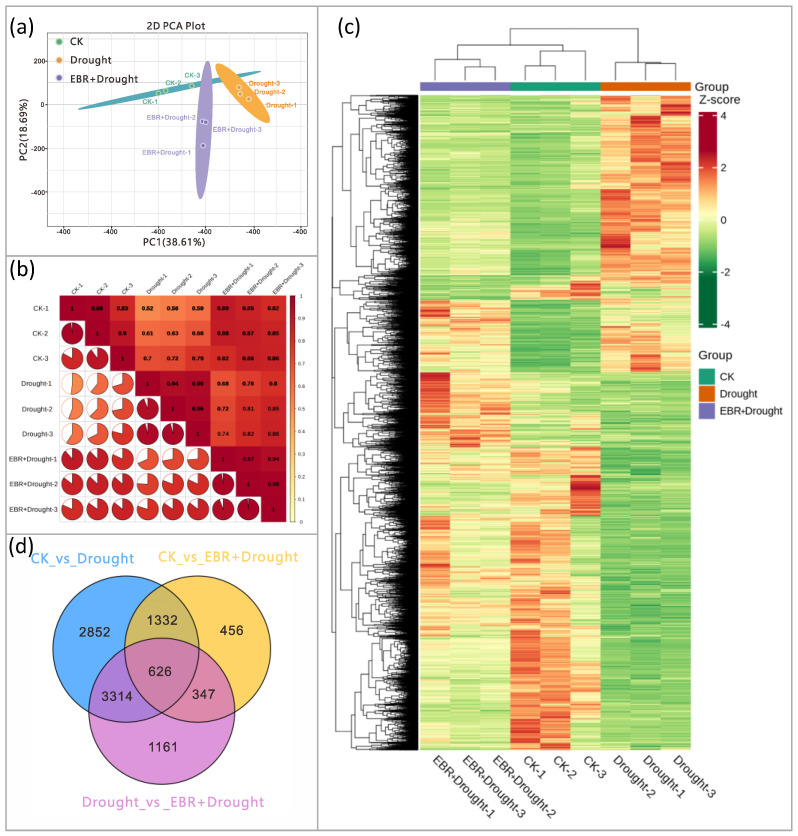
PCA analysis (**a**,**b**), cluster analysis (**c**), and Venn diagram related to DEGs (**d**). (**a**) PCA diagram. (**b**) Sample correlation heat map, (**c**) the horizontal axis represents sample names and hierarchical clustering results, and the vertical axis represents DEGs and hierarchical clustering results. The red color indicates high expression, and the green color indicates low expression. (**d**) Nonoverlapping regions represent DEGs that are specific to the different subgroups, and overlapping regions represent DEGs that are common to the different subgroups.

To explore the eigengenes that contributed substantially to drought stress under EBR treatments, we performed RNA-Seq analyses of quinoa seedlings at 6 d of drought stress. After removing the low-quality data, there were a total of 62.93 GB of clean data, obtained from nine samples. All Q20 and Q30 base values were greater than 98% and 92%, and GC contents were greater than 43%, indicating that the quality of the sequencing data was reliable and could be used for further analysis. PCA analysis can bring together samples with similar gene expressions, with closer proximity indicating greater sample similarity. The PCA results showed that the treatment and control samples were far apart from each other, indicating that there were significant differences in gene expression between the treated and control samples (Figure 6a). In addition, the correlation heat map showed that the correlation between biological replicates of the same group of samples was relatively high, indicating better reproducibility within the sample group (Figure 6b).

The DEGs in the quinoa seedlings at 6 d after drought stress were analyzed. There were three DEG comparison groups, among which there were 5448 genes (3353 up-regulated genes and 2095 down-regulated genes) in the Drought and EBR/Drought comparison group. In the CK and EBR + Drought comparison group, there were 2761 genes (1450 up-regulated genes and 1311 down-regulated genes). In the CK and Drought comparison group, there were 8124 genes (3138 up-regulated genes and 4986 down-regulated genes) (Supplementary Materials Figure S1). A differential gene clustering heat map showed that EBR treatment was able to significantly alter gene expression patterns under drought stress (Figure 6c). The Venn diagram shows 626 DEGs in common in the three groups and 2852, 456, and 1161 specific DEGs in the three comparison groups, respectively (Figure 6d).

**Figure 7:**
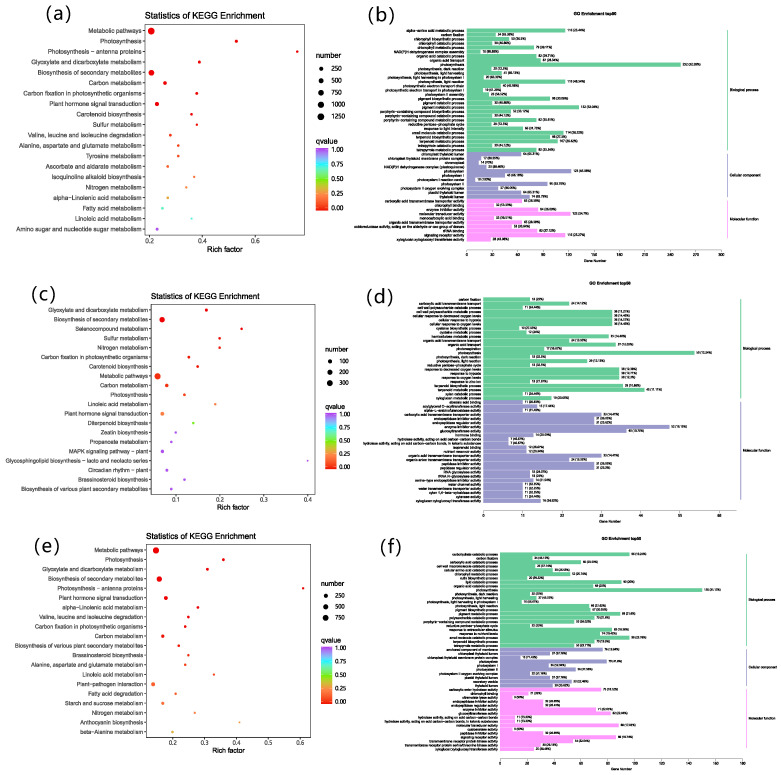
A KEGG enrichment analysis of DEGs (**a**,**c**,**e**), and a gene ontology (GO) histogram of DEGs (**b**,**d**,**f**). (**a**,**b**): CK and Drought; (**c**,**d**): CK and EBR + Drought; (**e**,**f**): Drought and EBR + Drought. The vertical coordinate indicates the KEGG pathway. The horizontal coordinate indicates the enrichment factor; the larger the enrichment factor, the higher the degree of enrichment. The larger the data point, the more DEGs enriched in the pathway. The redder the color of the data point, the more significant the enrichment (**a**,**c**,**e**). The horizontal coordinate indicates the number of differential genes annotated to the entry, and the vertical coordinate indicates the name of the GO entry. The numbers in the figure indicate the number of differential genes annotated to the entry, the specific value of the ratio of the number of differential genes annotated to the entry to the number of background genes annotated to the pathway is shown in parentheses, and the labels on the far-right side represent the classification to which the GO entry belongs (**b**,**d**,**f**).

To verify the biological functions of the differential genes induced by EBR treatment under drought stress, we conducted KEGG and GO enrichment analysis. A KEGG pathway analysis showed that the pathways significantly enriched in the three comparison groups included metabolic pathways, the biosynthesis of secondary metabolites, plant hormone signal transduction, and carbon metabolism (Figure 7a,c,e). When Drought is compared to EBR + Drought, the number of DEGs in metabolic pathways, the biosynthesis of secondary metabolites, plant hormone signal transduction, and brassinosteroid biosynthesis pathways increases in EBR + Drought. The classification of DEGs by GO analysis is to assess the DEG enrichment for molecular function, the cellular component, and the biological process (Figure 7b,d,f, and Supplementary Materials Figure S2). The GO analysis showed that in the CK and Drought comparison groups, up-regulated DEGs were mainly involved in photosynthesis, the photosystem, and molecular transducer activity (Figure 7d). In CK and EBR + Drought comparison groups, up-regulated DEGs were mainly involved in photosynthesis and enzyme inhibitor activity (Figure 7e). In the Drought and EBR + Drought comparison groups, up-regulated DEGs were mainly involved in photosynthesis, carbohydrate catabolic process, and the small molecule catabolic process (Figure 7f). The GO analysis and results showed that quinoa seedlings enhanced drought tolerance through enhanced photosynthesis, enzymatic antioxidant activity mechanisms, cellulose, secondary metabolite biosynthesis, plant hormone signal transduction, and carbon metabolism, enabling quinoa survival in drought stress.

**Figure 8:**
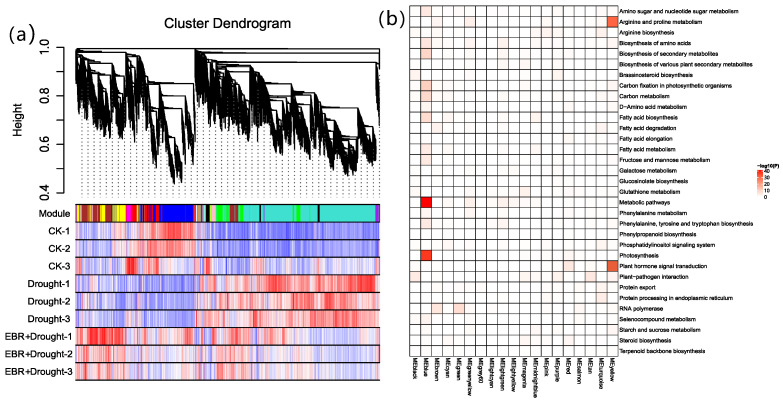
Co-expression modules constructed by weighted correlation network analysis. (**a**) Hierarchical cluster tree showing 19 modules with co-expressed genes. Branches represent positively correlated eigengene groups that are modules whose expression profiles should be merged due to their similarity. The figure shows the heat map of expression under drought stress. Every color denotes one specific coexpression module, and branches above stand for genes. (**b**) Gene ontology functional categories enriched in differential co-expression modules.

To identify regulatory networks related to BR biosynthesis and explore differences in genes, a weighted correlation network analysis (WGCNA) was performed to classify these DEGs into 19 modules (Figure 8a). The genes of the Mblue, Mbrown, Mgreen, and Myellow modules were transcribed at an increased level under the EBR + Drought treatment and were referred to as ‘drought-responsive modules’. And the Myellow module was enriched with GO terms related to ‘proline metabolism’ and ‘phytohormone signaling’ (Figure 8b). The results showed that these characterized genes collectively provided substantial contributions to drought stress response.

### 3.7. Expression Patterns of CqBIN2 and Proline Biosynthesis Genes in Quinoa Seedlings

**Figure 9:**
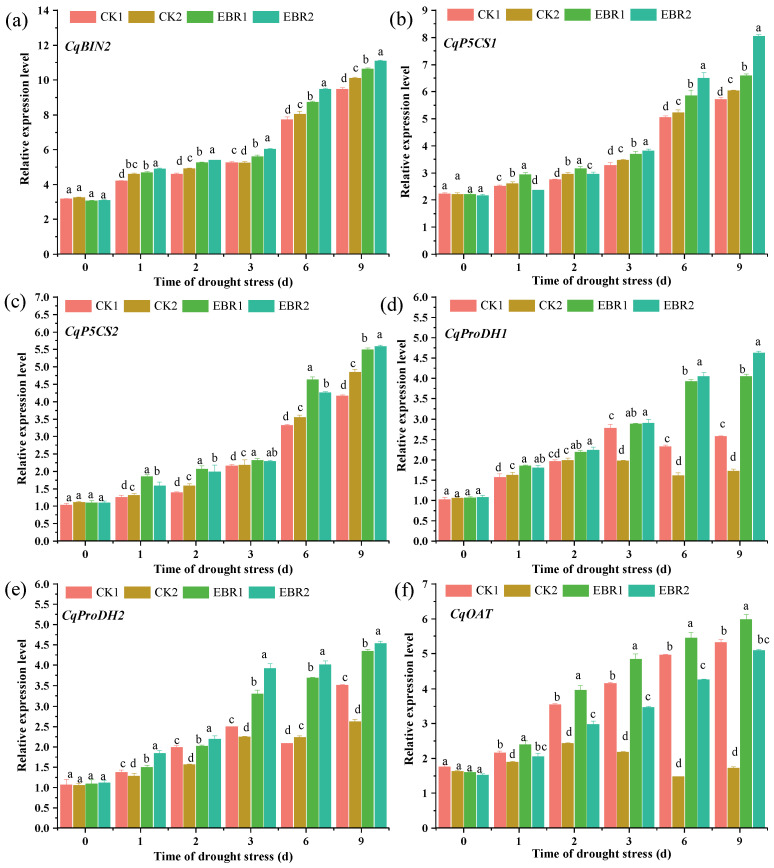
Effects of drought stress on the expression level of the *CqBIN2* (**a**) and proline biosynthesis genes *CqP5CS1* (**b**), *CqP5CS2* (**c**), *CqProDH1* (**d**), *CqProDH2* (**e**), and *CqOAT* (**f**). Values are means ± SD (*n* = 3); different lowercase letters indicate significant differences between treatments (*p* < 0.05, Duncan’s multiple range test). CK1: normal watering; CK2: drought stress; EBR1: EBR + normal watering; EBR2: EBR + drought treatment.

The effects of drought and EBR treatments on the mRNA levels of *CqBIN2*, *CqP5CS1*, *CqP5CS1*, *CqproDH1*, *CqproDH2*, and *CqOAT* were determined by RT-qPCR to detect highly expressed genes responding to drought signaling pathways. Under CK1 treatment, the expression level of *CqBIN2* increased by 6.06~12.82% in EBR1 plants. Drought stress markedly increased the expression level of *CqBIN2*, by 5.69~15.24% in EBR2 plants from 1 d to 9 d, compared to CK2 plants (Figure 9a). Proline biosynthesis gene expression levels exhibited significant differences between normal watering and drought stress in response to EBR treatment. The results of the expression levels of the *CqP5CS1* and *CqP5CS2* genes showed an increasing trend from 1 to 9 d under different treatments (Figure 9b,c). The highest level of *CqP5CS1* expression was observed at 25.02% at 9 d in EBR2 plants compared to CK2 plants (Figure 9b). Furthermore, the highest expression level of *CqP5CS2* was observed in EBR2 plants at 2 d, which increased by 20.17% compared to CK2 plants (Figure 9c).

**Figure 10:**
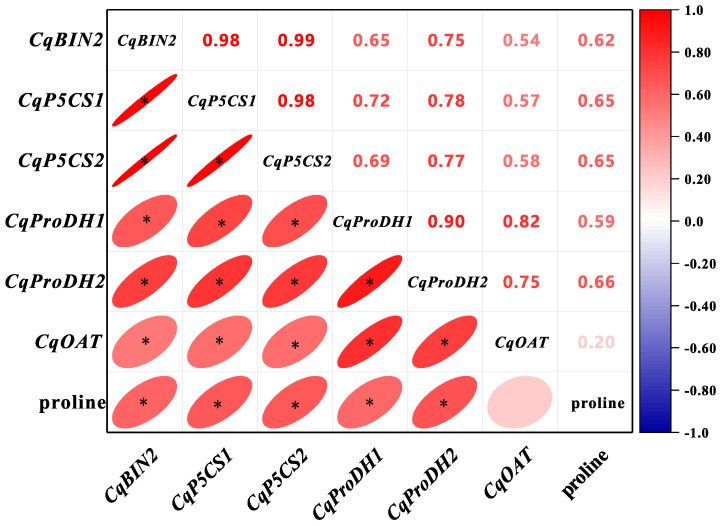
The Pearson correlation coefficient of *CqBIN2* and the proline biosynthesis genes, with significance denoted by the * symbol (*p* < 0.05).

Compared to CK2 plants, the expression level of *CqproDH1* was increased by 60.20% and 62.79% in EBR2 plants at 6 d and 9 d, respectively. There were no significant differences in the expression level of *CqproDH1* in both EBR1 plants and EBR2 plants from 1 to 3 d (Figure 9d). Additionally, the expression level of *CqproDH2* increased significantly by 28.47~44.36% in EBR2 plants over CK2 plants from 1 to 9 d and reached the maximum value of 44.36% at 6 d (Figure 9e). There was a significantly upward trend in the expression level of *CqOAT* both in normal watering and in EBR treatment (Figure 9f). The expression level of *CqOAT* was significantly increased by 7.69%~66.34% from 1 to 9 d and reached the maximum value at 9 d in EBR2 plants compared to CK2 plants. In particular, there was no significant difference in the expression level of *CqOAT* between CK1 plants and EBR2 plants at 1 d and 9 d (Figure 9f). These results indicated that the up-regulated expression level of the *CqBIN2* and proline biosynthesis genes may be responsible for the EBR-induced accumulation of proline, which enhanced drought tolerance in quinoa seedlings. The Pearson correlation coefficients (PCC) were calculated based on the expression levels of CqBIN2 and proline biosynthesis genes (*CqP5CS1*, *CqP5CS2*, *CqProDH1*, *CqProDH2,* and *CqOAT*) as well as the accumulation of proline under different treatments. The larger the absolute value of the PCC was, the stronger the correlation, as shown in Figure 10. The expression levels of *CqBIN2* and the proline biosynthesis genes were positively correlated with the accumulation of proline. Additionally, except for *CqOAT*, all were significantly correlated with proline (*p* < 0.05) (Figure 10). These results indicated that the up-regulated expression level of *CqBIN2* and proline biosynthesis genes may be responsible for the EBR-induced accumulation of proline, which enhanced drought tolerance in quinoa seedlings.

### 3.8. Silencing of CqBIN2 Improves Drought Tolerance in Quinoa

**Figure 11:**
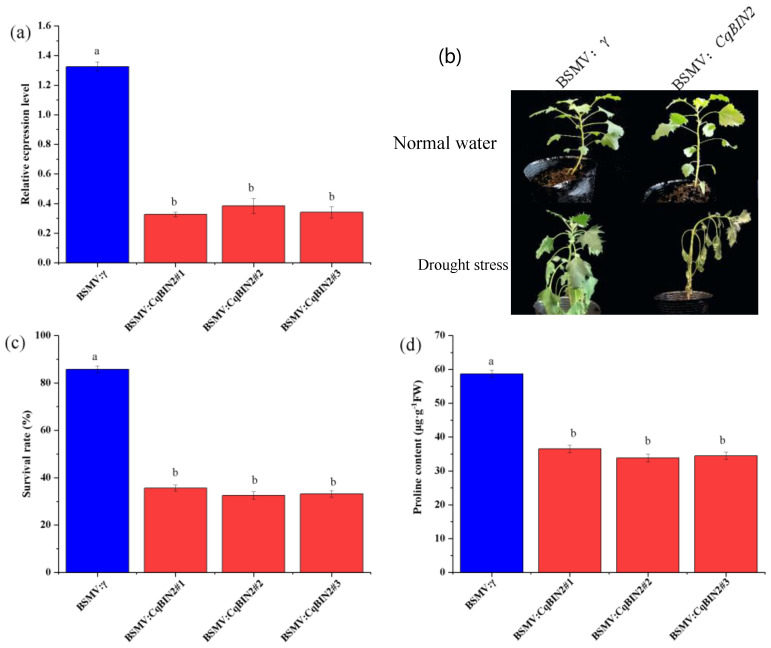
Drought tolerance of BSMV: *CqBIN2* silenced the quinoa seedling. (**a**) Analysis of transient silencing efficiency of BSMV: *CqBIN2*. (**b**) Drought-tolerant phenotypes of BSMV: *CqBIN2* silenced the quinoa cv. Fielder. (**c**) Statistical analysis of survival rates. (**d**) Proline content in the leaves of BSMV: γ controls and BSMV: *CqBIN2* silenced plants. Values are means ± SD (*n* = 3); different lowercase letters indicate significant differences between treatments (*p* < 0.05, Duncan’s multiple range test).

Considering that *CqBIN2* is responsible for enhancing drought tolerance, the BMSV: *CqBIN2* plants were generated in quinoa cv Fielder by BMSV-VIGS. Under well water, the expression levels of *CqBIN2* were significantly reduced in BMSV: *CqBIN2* plants by 71~75%, compared to BMSV: γ controls (Figure 11a). In well water, there were no phenotype differences between BMSV: *CqBIN2* and BMSV: γ controls. However, the survival rate was significantly reduced to 58~62% while the BMSV: γ controls survival rate was 85% (Figure 11b,c) after withholding for 20 d followed by a 3 d recovery period with full irrigation. Proline is an important substance involved in osmoregulation in plants and plays an important role in mitigating abiotic stress responses in plants. In the present study, proline content was determined and found that it was significantly reduced in BMSV: *CqBIN2* plants than in BMSV: γ controls (Figure 11d). Therefore, we suspected that EBR treatment might promote drought tolerance by up-regulating the *CqBIN2* expression level.

## 4. Discussion

Water scarcity has become a global problem that needs to be solved. Drought stress is an abiotic stress that seriously affects plant growth, development, and other life activities and has become a key factor constraining the sustainable development of agriculture [22,23]. Brassinosteroids participate in cell elongation and control plant growth and development processes, such as cell division [24]. The application of EBR could significantly improve plant tolerance to drought stress. Compared to normal watering (CK1), drought stress (CK2) could significantly reduce root morphology parameters (Figure 1). The reduction in root morphology parameters was due to the inhibition of cell division and expansion under drought stress, resulting in the inhibition of growth and biomass accumulation. EBR application could alleviate the decrease in the root morphology parameters; it may be due to EBR treatment up-regulating the expression level of genes that promote root development [6,7].

Drought stress drastically affected the lipid metabolism, leading to excessive ROS production and lipid peroxidation. The accumulation of ROS and MDA leads to oxidative damage to plant cells, tissues, and organs under abiotic stress [25,26]. EBR treatment significantly reduced the H_2_O_2_, $O_{2}^{∙-}$, and MDA contents, which is in line with Rayyan et al. [27], who reported that the application of EBR could improve drought tolerance in tobacco by strengthening osmoregulation to scavenge reactive oxygen species, which indicated that EBR is essential to reduce the overproduction of ROS under drought conditions. EBR treatment could significantly increase antioxidant enzyme activity (including SOD, CAT, POD, APX, GR, MDHAR, and DHAR) and osmolyte content (soluble protein and proline), which is supported by the up-regulated expression level of related genes in EBR-treated seedlings. Previous research also reported that BR is involved in the regulation of antioxidant enzyme activities and scavenged ROS under drought stress [28,29]. Furthermore, EBR treatment could up-regulate the expression level of *ZmBZR1* and *ZmBES1*, which induced the expression of genes encoding antioxidant enzymes and thus positively regulated drought stress in maize [29]. In addition to higher antioxidant enzyme activities and osmolyte content, EBR application also increases the transcript levels of proline biosynthesis genes (including *CqP5CS1*, *CqP5CS1*, *CqproDH1*, *CqproDH2*, and *CqOAT*), resulting in the accumulation of proline to act against the oxidative stress in response to drought stress [30]. Moreover, it also suggests that the EBR-induced accumulation of proline, which acts as an osmoprotectant, facilitates the alleviation of drought damage to cells in quinoa seedlings. Xia et al. [6] reported that EBR treatment up-regulated the expression level of the proline synthesis gene *P5CR1* and down-regulated the expression of the proline degradation gene *ProDH*, causing the accumulation of proline and alleviating drought stress in kiwifruit seedlings. These findings validated previous studies that proline accumulation could help plants reduce water loss under drought stress. Previous researchers reported that proline biosynthesis was regulated by multiple transcription factors [22,31]; however, the molecular mechanism of BRs that mediates proline biosynthesis is unclear as to how it is related to drought resistance.

Previous research suggested that there exists a complex network between brassinosteroids and other hormones in the regulation of drought resistance [25,32,33]. The results showed that EBR application could increase the endogenous ABA contents under drought stress, which may be due to EBR application activating the expression of ABA biosynthesis genes [34,35]. This can also be supported by Yuan et al. [36] , who reported that applications of exogenous EBR significantly increased ABA content in tomatoes under drought stress. Therefore, ABA plays a crucial role in plant tolerance to abiotic stress, which could strengthen antioxidant enzyme activity, scavenging ROS, soluble protein, and proline content and up-regulate the expression level of proline biosynthesis. In addition, in BR signaling, BIN2 and its homologs play an active role in the ABA primary signaling pathway and control the ABA signaling output in response to abiotic stress, and the ABI1-BIN2-RD26 regulatory module was shown to play an important role in the response to drought stress [17,37]. Meanwhile, EBR application increases endogenous BR content, according to Nie et al. [38], who reported that exogenous EBR application improves drought resistance in tomatoes attributed to a significant increase in BR content and BR signaling intensity by EBR application.

The higher BR content may be due to the fact that EBR treatment significantly increases the expression level of *CqBIN2* under drought stress (Figure 9a). BIN2 and its homologs are highly conserved serine/threonine kinases that participate in the regulation of many developmental and abiotic stress response pathways [20,39,40]. Previous research reported that BIN2 phosphorylates to positively regulate drought tolerance [17,18], and BIN2 might regulate H_2_O_2_ production in BR signaling possibly through direct phosphorylation [41]. The overexpression of *GmBIN2* significantly increases proline and superoxide dismutase activities and improves drought tolerance in *Arabidopsis* and soybeans [37]. In the present study, *CqBIN2* was silenced in quinoa seedlings, whose results showed that survival rate and proline content were significantly reduced under drought stress (Figure 11), which indicated that it has the potential to regulate growth and ROS scavenging under drought stress. Hence, we hypothesize that CqBIN2 exerts a strong regulatory role in improving plant resistance to a water deficit. It is also a complex molecular mechanism that still needs further research.

## 5. Conclusions

In summary, the research demonstrated that the application of EBR mitigates drought damage to quinoa seedlings. The application of EBR improved the roots morphology parameters, soluble protein and proline contents, antioxidant enzyme activity, and ABA and BR contents, while decreasing the H_2_O_2_, $O_{2}^{∙-}$, and MDA contents to maintain plant growth. Meanwhile, EBR treatment could up-regulate the expression level of the *CqBIN2* and proline biosynthesis genes (*CqP5CS1*, *CqP5CS1*, *CqproDH1*, *CqproDH2*, and *CqOAT*) to promote proline accumulation, ultimately improving the drought resistance of quinoa seedlings. In the present study, it seems that EBR treatment induced the expression of *CqBIN2* to activate the expression of the proline biosynthesis gene to enhance proline accumulation and improve the drought resistance of the quinoa seedlings.

## Data Availability

Data are contained within the article and Supplementary Materials.

## Supplementary Materials

The following supporting information can be downloaded at: https://www.mdpi.com/article/10.3390/plants13060873/s1, Table S1. Primers sequences for RT-qPCR. Table S2. Primers sequences for BSMV-VIGS. Figure S1. The diagram of differential gene volcano and differential gene statistics (a): CK and Drought; (b): CK and EBR + Drought; (c): Drought and EBR + Drought; (d): Differential gene statistics chart. Figure S2. 2 GO enrichment scatter plot and Go classification histogram of DEGs (a,b): CK and Drought; (c,d): CK and EBR + Drought; (e,f): Drought and EBR + Drought.
