# Supplementary material for: Exogenous 24-Epibrassinolide Enhanced Drought Tolerance and Promoted BRASSINOSTEROID-INSENSITIVE2 Expression of Quinoa
Source: Plants (Basel). 2024 Mar 18;13(6):873. doi: 10.3390/plants13060873 (PMC10974127; doi:10.3390/plants13060873)
Supplement: Supplementary file 1 [file plants-13-00873-s001.zip › plants-2855047-supplementary.pdf]

Supplementary Materials

Table S1. Primers sequences for RT-qPCR.

Gene name		Primers sequences
CqBIN2	**F**	GTGATGGCTTGGCTGAGAGGTG
	R	CGGTCGGAGATTGGAAGTAGAGTC
CqP5CS1	F	AATGCCTAATACTCCTGCTGCTGTG
	R	GCCTTGCCAACTGCTCCAAATAATC
CqP5CS2	F	AATGCCTAATACTCCTGCTGCTGTG
	R	GCTCGCCATCCTCTTCAGTTGC
CqProDH1	F	AAGTTGGCTGCTTCACTAGGGTTTG
	R	CGACATTATGGGTGGCAAGGACTG
CqProDH2	F	ATTGGCTGCTTCACTTGGGTATGAG
	R	CGACATTATGGGTGGCAAGGACTG
CqOAT	F	AGCCTACCCACAACACAATCATAAG
	R	CTTCAGACAGTGCCTTGGTTCC
CqMON1	F	GTATTGGTGGTCCTTGTG
	R	CTGCTGTGGTGTATTGATT

Table S2. Primers sequences for BSMV-VIGS.

Gene name		Primers sequences
CqBIN2	**F**	TAGAAGGCCTCCATGATGGAGCAAGGGGTTTCCTC
	R	GAGACGCGTGAGCTCAAGCTGCTCAACTTCAGCGT

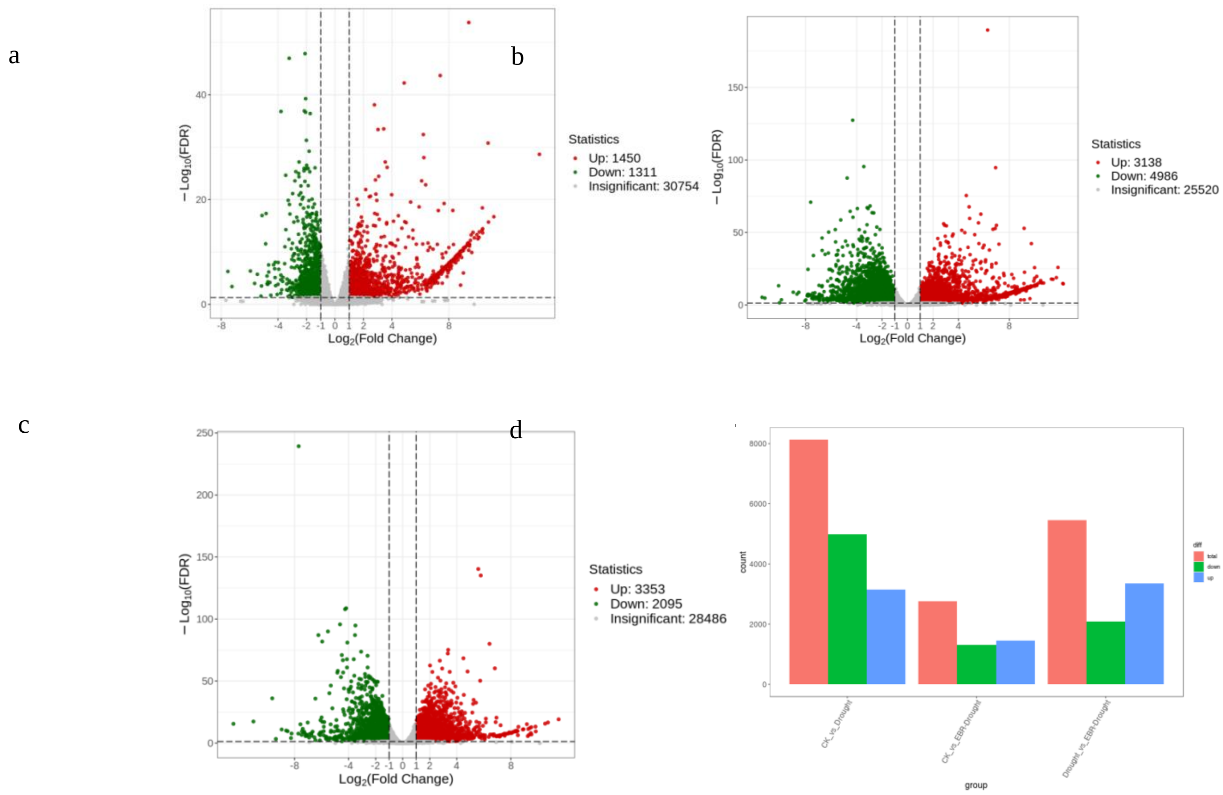

Figure S1. The diagram of differential gene volcano and differential gene statistics (a): CK and Drought; (b): CK and EBR + Drought; (c): Drought and EBR + Drought; (d): Differential gene statistics chart.

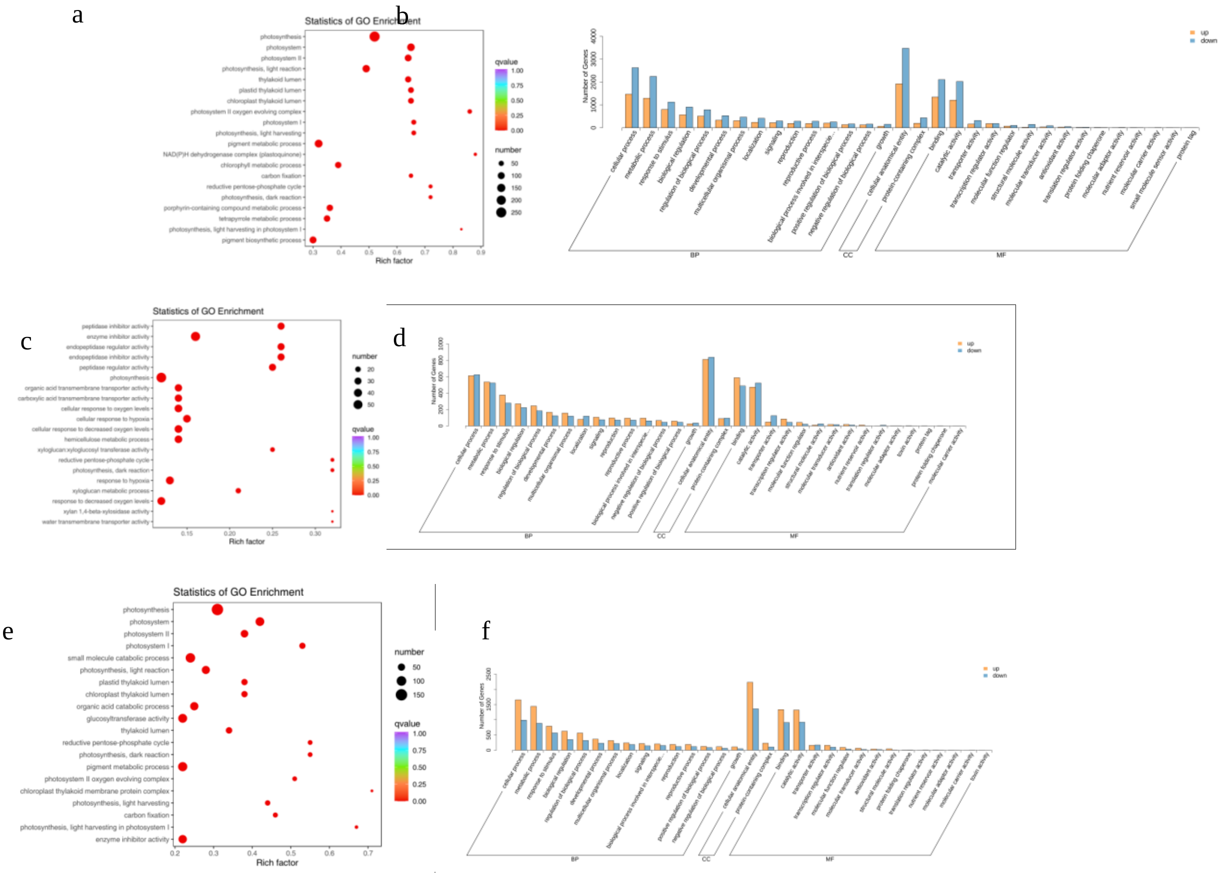

Figure S2. 2 GO enrichment scatter plot and Go classification histogram of DEGs (a, b): CK and Drought; (c, d): CK and EBR + Drought; (e, f): Drought and EBR + Drought.
